# Post-Harvest Behavior of Seedless Conical and Mini-Conical Peppers: Weight Loss, Dry Matter Content, and Total Soluble Solids as Indicators of Quality and Commercial Shelf-Life

**DOI:** 10.3390/foods13121889

**Published:** 2024-06-16

**Authors:** Manuel Díaz-Pérez, José Javier Hernández-García, Ángel Carreño-Ortega, Borja Velázquez Martí

**Affiliations:** 1 Department of Engineering, University of Almería, Research Centre CIAIMBITAL, Agrifood Campus of International Excellence (CeiA3), La Cañada de San Urbano, 04120 Almería, Spain; jhg173@inlumine.ual.es (J.J.H.-G.); acarre@ual.es (Á.C.-O.); 2 Departamento de Ingeniería Rural y Agroalimentaria, Universitat Politècnica de València, Camino de Vera s/n, 46022 Valencia, Spain; borvemar@dmta.upv.es

**Keywords:** *Capsicum annuum*, dry matter, total soluble solids, storage, marketing probability, seedless fruit

## Abstract

This study aimed to assess the post-harvest dynamics of seedless conical and mini-conical pepper cultivars in terms of fruit weight loss, dry matter content, and soluble solid content. The above parameters were demonstrated to be effective commercial pepper shelf-life indicators. The commercial quality of pepper fruit intended for export was evaluated weekly under simulated fruit storage conditions for over 28 d. Results revealed that fruit weight loss, dry matter content, and soluble solid content were affected by cultivar type and storage duration. Additionally, a strong correlation between these variables was observed confirming their linear relationship which was more profound between dry matter and total soluble solid content. Daily changes during storage were similar in both seedless conical and mini-conical peppers, while the fruit weight loss daily rate was greater than that of dry matter. Water loss was identified to be the main factor causing reduced fruit quality. Solid content reduction occurred predominately during the initial storage period. Notably, fruit with lower dry matter content at harvest tended to maintain their commercial quality for a longer time due to their ability to resist water loss without any visible signs of deterioration, which is beneficial during prolonged storage.

## 1. Introduction

In a world facing challenges such as food security, resource sustainability, and efficiency, the problem of food loss along the distribution chain has raised growing societal concerns [1]. Globally, 14% of the production is lost between harvest and distribution, corresponding to approximately USD 400 billion [2] in revenue. Another 17% is wasted during distribution and after reaching the final consumers [3]. A large proportion of losses related to fruit and vegetables occur from harvest to consumption [4]. Thus, improving the commercial life of products during the post-harvest stage is crucial.

Due to the culinary and medicinal properties of pepper, the global fresh pepper market is experiencing significant growth [5]. However, identifying the factors that cause deterioration in fruit quality is crucial to ensure prolonged post-harvest shelf-life [6]. A relatively reduced shelf-life of pepper fruit intended for fresh consumption can be caused by various abiotic and biotic factors [7]. These factors include mechanical damage, the presence of insects or diseases, moisture loss due to transpiration and respiration, exposure to extreme temperatures (e.g., freezing or dehydration), physiological disorders, such as cold symptoms induced by low temperatures during storage, and dry matter losses caused by respiration [8].

Even a small decrease in the water content of pepper fruit leads to the loss of inherent freshness and firmness, which results in a reduction in their quality, durability, and market value [9]. Water loss during storage results in weight loss in pepper fruit [10] and in combination with putrescence are the main post-harvest problems of pepper. Multiple factors influence weight loss during storage, including relative humidity (RH), ventilation, packaging, temperature, and fruit size [11], with water loss during the post-harvest period being responsible for both qualitative and quantitative losses. Excessive dehydration leads to dull skin and shrunken, wrinkled fruit, with the severity of these symptoms varying based on the specific plant variety and storage conditions [8]. Pepper fruits are hollow with a high surface-area-to-fresh-weight ratio and, therefore, lack the internal water reserves typical of solid fruit, such as tomatoes and apples, making them highly susceptible to water loss (shriveling). Small amounts of water loss can lead to decreased turgidity and firmness, impacting fruit quality, shelf-life, market value, and perceived freshness. The extent of water loss from the fruit typically determines the post-harvest quality of peppers [12].

Identifying easy and quick-to-measure indicators is useful for characterizing quality, enhancing shelf life, and reducing waste in peppers. Color, texture, and microbiological analysis are employed in various countries as indicators of pepper quality and shelf life. Some of these indicators use CO_2_ indicator labels based on a pH-sensitive dye containing methyl red and bromothymol blue to measure the freshness of peppers [13]. These labels change color when the pH varies due to an alteration in the concentration of carbon dioxide in the packaging, which is associated with the deterioration of peppers [14]. Additionally, Hernández-Carrión et al. [15] demonstrated that texture could be a reliable indicator of pepper quality and shelf life.

Supply must be increased, and losses and waste reduced to satisfy the rising food demand in the upcoming years [16]. Recent research has focused on extending the shelf-life of peppers after harvest. Among these studies, cultivar genetics has been identified as a promising approach to enhance the commercial durability of peppers [17]. By incorporating genes into new crop varieties to improve post-harvest characteristics, it is possible to reduce losses and wastage, especially along extended distribution networks [18]. Additionally, identifying easily implemented markers that enable selecting phenotypes with greater potential to increase commercial longevity is crucial.

Currently, studies on the post-harvest characteristics and essential changes that occur in conical parthenocarpic peppers during prolonged storage are absent. Addressing this lack of knowledge is the main interest of our study. This study mainly aimed to gain a deeper understanding of the fruit weight loss process, dry matter content, and soluble solid content during the post-harvest phase in seedless conical peppers. Additionally, this study investigated the potential of these parameters to be used as indicators of the probability of successful pepper marketability. In previous studies, researchers have confirmed the effectiveness of the above-mentioned quality and commercial longevity markers in fruits like tomatoes [19] and cucumbers [20]. However, the applicability of these markers in parthenocarpic pepper fruit remains unexplored.

## 2. Materials and Methods

### 2.1. Vegetal Material

Seedless conical pepper phenotypes grown in greenhouses in southeastern Spain (Almería, Spain) were studied during the 2022–2023 growing season to understand the post-harvest behavior of parthenocarpic peppers during prolonged storage.

‘Conical-type’ peppers are characterized by their fine flesh, conical shape, and pointed ends. Currently, two different types of seedless conical peppers exist that differ in size, i.e., the “mini-conical” peppers, which are 7–8 cm in length and 3.5–4 cm in width, and the “conical” peppers, which are 10–11 cm in length and 3.5 cm in width. These phenotypes are usually characterized by red, orange, or yellow fruit coloration, and their fruit weight varies depending on their wall thickness. Herein, two types of seedless conical pepper phenotypes (‘109’ and ‘563’) and three ‘mini-conical’ seedless phenotypes (‘SL001’, ‘SL002’ and ‘SL003’) were evaluated. The ‘109’ and ‘563’ phenotypes were red while the ‘SL001’, ‘SL002’, and ‘SL003’ phenotypes were red, orange, and yellow, respectively.

The evaluated fruit samples were obtained from professional producers who grew and exported peppers. Seedlings were transplanted in the first week of August 2022 and cultivated using the typical agronomic practice for pepper cultivation. The fruits were harvested at the optimal maturity level for sale. We harvested the peppers at full development and optimal maturity, as indicated by the characteristic shape and color of each cultivar. The harvesting was carried out manually using scissors to cut above the fruit, leaving a small peduncle attached. Additionally, the fruits were handled carefully in field containers to prevent bruising and damage.

### 2.2. Experimental Design

Experiments followed a split-plot design, which is a factorial design where complete sampling randomization is impossible [21]. The variability factors examined were as follows: Factor A, which included different parthenocarpic pepper phenotypes (‘SL001’, ‘SL002’, and ‘SL003’ for the mini-conical type; and ‘109’ and ‘563’ for the conical type) and Factor B, which included storage times of 0, 7, 14, 21, and 28 d. The study was replicated during the main production months (Blocks: February, March, April, and May). Each block (study month) was divided into plots (phenotypes), which were subsequently divided into subplots (storage times).

Samples consisting of 100 fruits were collected for each cultivar and month and evaluated by simple random sampling to obtain representative samples. The samples were obtained from professional growers who cultivated peppers for export in European countries. The samples were collected from plants that were cultivated using standard agronomic practices employed in the area. The selected fruit were harvested at the optimal state of commercial maturity and were free of defects, which guaranteed that they were suitable for export.

The collected samples were labeled and sent for analysis to a laboratory specializing in food quality tests at the University of Almeria (Almeria, Spain). Each sample was divided into two subsamples: one consisted of 25 fruit used to evaluate the commercial quality at the time of harvest, and the other of 75 fruit used to evaluate the quality at 7-day intervals, i.e., at 0, 7, 14, 21, and 28 days of storage. During storage, we simulated the environment that the fruit would experience from harvest to reaching the consumer. This period included an initial stage of seven storage days in a cold room at 10 °C, a relative humidity between 85% and 95% under dark conditions to simulate transit during refrigerated transport and storage by wholesalers, intermediaries, and supermarkets. These conditions followed the recommended standards for pepper transportation, storage, and distribution [22,23]. During the following 7-to-28-day storage period, the fruit were stored in a chamber at room temperature (18–20 °C), simulating the period of fruit display and sale to consumers.

Several parameter measurements were performed in the laboratory for each fruit at harvest time, including total soluble solid content (TSS, %) and dry matter content (DMC_i_, %). An ATAGO PR-101α digital refractometer (ATAGO Co., Ltd., Tokyo, Japan) with a measurement range of 0–85% and an accuracy of 0.1% was used to determine the fruit TSS content. The equipment was calibrated using distilled water. A fragment of approximately 1 g taken from the central area of the fruit was used to obtain the necessary juice, and a metal press equipped with a sieve containing small holes was used to collect the liquid used for measurement. The remaining fruit was used to determine the DMC_i_ content.

The parameters evaluated for each of the 75 stored fruit samples included the loss of commercial quality, weight loss (%), total soluble solid TSS (%), and DMC_i_ (%) content. The TSS and DMC_i_ were determined in two subsamples of 25 fruit each after 14 and 28 days of storage, respectively.

The DMC_i_ was determined following the method used by Valverde-Miranda et al. [20], in which the samples were dried at 70 °C until the weight variations between consecutive measurements made every 2 h were <0.1% [24]. DMC_i_ was calculated as a percentage of the fresh weight of the fruit sample at the time of measurement (0, 14, or 28 storage days), as described in Equation (1).

(1) $${DMC}_{i}\;(\%)=\frac{{DV}_{i}}{{FW}_{i}}\times 100$$

where ${DMC}_{i}$ is the percentage of the fruit dry matter for times i = 0, 14, and 28 storage days, ${DV}_{i}$ is the dry weight of the fruit sample, and ${FW}_{i}$ is the fresh weight of the fruit sample.

The percentage of dry matter that the stored fruit would have at the time of harvest was calculated to determine the fruit dry matter loss during storage, assuming that any weight loss during storage was solely due to water loss. This value was compared to the percentage of fruit dry matter at harvest. The original dry matter content (DMC_0_) in relation to the fresh weight of the fruit sample at harvest was estimated from the fruit sample weight at the time of measurement by adding the corresponding percentage of weight loss observed after 14 or 28 days of storage. This calculation is detailed in Equation (2).

(2) $${DMC}_{0}\;(\%)=\frac{{DV}_{i}}{\left({FW}_{i}+{PP}_{i}\right)}\times 100$$

where ${DMC}_{0}$ is the dry matter percentage of the fruit at the harvest time, assuming that any weight loss during storage was solely due to water loss. ${DV}_{i}$ denotes the dry weight of the fruit sample, ${FW}_{i}$ refers to the fresh weight of the fruit sample before drying, and ${PP}_{i}$ indicates the weight loss of the fruit sample at the time of measurement i (when i = 0, ${PP}_{i}=0,$ and ${DMC}_{i}={DMC}_{0}$).

The loss in commercial quality of the fruit was individually evaluated during the storage period, considering decay, presence of rot caused by microorganisms (mainly bacteria and fungi), and damage caused by cold conditions. These factors are widely recognized as the main causes of the loss in peppers’ commercial value along the distribution chain [17]. Based on the evaluation of these parameters, each fruit was classified as commercially or non-commercially viable depending on the level of deterioration degree observed during the storage period. The incidence of rot caused by fungi and bacteria was very low, so we analyzed the total damage caused by pathogens without differentiating the type of pathogen.

Fruit weight loss is the main indicator of post-harvest water loss [25]. Therefore, weight loss was evaluated for each of the 75 fruit subsamples during the storage period by measuring fruit weight at 7-day intervals from the time of harvest. We used a RADWAG Wagi Elektroniczne scale, model PS 600.R2, with a maximum capacity of 600 g and sensitivity of 0.01 g. Weight loss was calculated as a percentage of the initial weight of each fruit, as described by Frans et al. [26].

### 2.3. Statistical Analysis

Data were subjected to an analysis of variance according to the split-plot design described in Equation (3):

*Y_ijks_* = *μ* + *α_i_* + *β_j_* + (*αβ*)*_ij_* + γ*_k_* + (*α*γ)*_ik_* + (*β*γ)*_jk_* + (*αβ*γ)*_ijk_* + ε*_ijks_* (3)

where i = February, March, April, and May; j = ‘SL001, ‘SL002’, and ‘SL003’ (for the mini-conical cultivar) and ‘109’ and ‘563’ (for the conical cultivar); k = 0, 7, 14, 21, and 28 storage days; s = 1, 2, …, 25 fruit. Additionally, Y_ijks_: is the ijks-th observation, µ: is the global mean, *α*_*i*_, *β*_*j*_, and (*αβ*)_*ij*_ represent the whole plot and correspond to the factor A (the month of study), the main treatments (factor B: the cultivar) and the whole plot error (*AB*), respectively; Υ_*k*_, (*α*Υ)_*ik*_, (*β*Υ)_*jk*_, and (*αβ*Υ)_*ijk*_ represent the whole subplot and correspond to the subplot treatment (factor C), interactions (*A* × *C* and *B* × *C*), and subplot error (*A* × *B* × *C*), respectively, while ε_ijkl_ is the experimental error [27].

Orthogonal contrast analyses were performed as described by Saville [28] to identify any linear or quadratic effects of fruit weight loss, dry matter content, and soluble solid content as a function of storage time.

Additionally, simple analyses of variance were performed, according to the model Y_ij_ = µ + α_i_ + ε_ij_, to study the evolution of fruit weight loss (%) in the commercially and non-commercially viable fruit as a function of the storage time and the fruit’s dry matter content at the harvest time as a function of the days in which the fruit ceased to be marketable. The normality and homoscedasticity hypotheses were verified in all analyses. The Bonferroni test was used to compare the means of each treatment.

Furthermore, correlation and simple linear regression analyses were performed using the least squares method. The significance of the linear model was evaluated using analysis of variance. Pearson’s correlation coefficient (r) and the coefficient of determination (R^2^) were used for analyzing goodness-of-fit. Additionally, normality, homoscedasticity, linearity, and autocorrelation absence were verified. Binary logistic regression analyses were performed according to the methodology described by Valverde-Miranda et al. [20].

IBM SPSS Statistics version 28 and Statgraphics Centurion 19 version 19.5.01 were used to perform data analyses.

## 3. Results

The appropriate experimental design of our study was a factorial design with multiple factors, but since complete randomization of the sampling order was impossible, the split-plot factorial design was selected for the study objectives. In the present study, we examined two different types of cultivars (two conical peppers and three mini-conical peppers). Samples were evaluated at five different time points during storage. The effects of these two factors on the commercial life of the fruit were evaluated. Furthermore, because the production period of this crop lasts for several months, we opted to replicate the experiment monthly during the main production months (February, March, April, and May) and considered each replicated month as a block.

### 3.1. Post-Harvest Evolution: Soluble Solids Content, Dry Matter Content and Weight Loss, and Marketability Probability

In our split-plot experimental design, Factor A (the cultivar) included different pepper phenotypes, with mini-conical types (‘SL001’, ‘SL002’, and ‘SL003’) and the conical types (‘109’ and ‘563’). Factor B represented storage time (0, 7, 14, 21, and 28 d). Each study month (block) was divided into plots (cultivars), and each plot was further subdivided into subplots (storage times). Table 1 indicates the analysis results based on the experimental design. 

Factor A (the cultivar) exhibited a significant effect on the fruit weight loss (FWL), DMC_i_, and TSS among the cultivars ‘SL001’, ‘SL002’, and ‘SL003’. Specifically, the fruit of cultivar ‘SL003’ showed the highest weight loss (23.5%) and the lowest dry matter (12.7%) and TSS (11.9%) content. In contrast, ‘SL002’ fruit exhibited the highest DMC_i_ (17.4%) and TSS (16.5%) contents and the lowest FWL (14.9%). Lastly, the fruit of ‘SL001’ exhibited moderate weight loss, dry matter, and soluble solids contents. As for the “conical” peppers, the differences between cultivars ‘109’ and ‘563’ were not significant. These results suggest that the greater the dry matter and soluble solid content of peppers, the lower is the weight loss of the fruit during storage.

A consistent pattern was observed in both pepper types in the effect of storage time: fruit weight loss increased with storage time. Furthermore, orthogonal contrast analysis showed a positive linear effect between these two parameters in both types of cultivars. This suggests that weight loss is an inherent long-term storage phenomenon in both pepper types. The average daily weight loss for the overall storage time was 1.09% in the “mini-conical” and 1.15% in “conical” peppers, although it varied along the different time intervals measured. The average daily weight loss for the first 7 d was approximately 0.64% for both pepper types. In the 7–14-day interval, it increased to approximately 1.03% in the “mini-conical” and 1.15% in the “conical” peppers. Finally, during days 21 to 28, the average daily weight loss increased by approximately 1.28% for both pepper types. As expected, water loss was observed as a natural consequence of long-term storage. The significant weight loss differences observed at different time intervals highlight the relevance of storage duration.

Furthermore, a highly significant interaction (***) was observed between cultivars ‘SL001’, ‘SL002’, and ‘SL003’ and storage time, indicating that the DMC_i_, TSS, and FWL of a particular cultivar may depend on the storage time, and vice versa. However, in cultivars ‘109’ and ‘563’, the only interaction observed was in the case of FWL. All cultivars showed an increasing trend towards weight loss, total solid content, and soluble solid content during storage, although differences existed in the magnitudes of the responses among the mini-conical cultivars. The DMC_i_, TSS, and FWL in ‘SL002’ were more prone to increase than in ‘SL001’ and ‘SL003’, which exhibited a similar and more moderate response (Table 1).

In general, DMC_i_ and TSS measured at 0, 14, and 28 d tended to increase with storage time in both pepper types and a significant positive linear effect was observed using orthogonal contrast analysis. This phenomenon reflects an increasing trend of total solids and soluble solids in relation to the total fruit content at the time of measurement.

**Figure 1:**
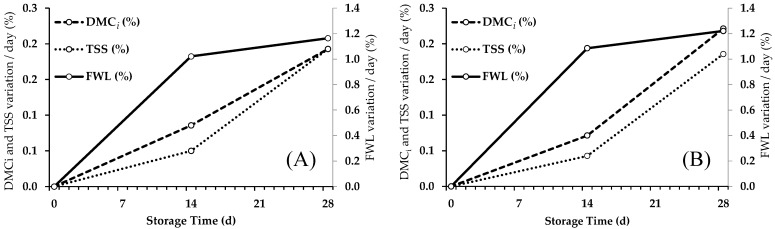
Average daily variation in the dry matter content (DMC_i_, %), total soluble solids content (TSS, %), and fruit weight loss (FWL, %) measured at 0, 14, and 28 days of storage for: (**A**) cultivars ‘SL001’, ‘SL002’, and ‘SL003’; (**B**) cultivars ‘109’ and ‘563’.

A similar behavior in the average daily variation was observed in both mini-conical (Figure 1A) and conical (Figure 1B) peppers concerning the percentage of FWL, DMC_i_, and TSS measured at 0, 14, and 28 d. A gradual daily weight loss was observed along with daily increases in dry matter and soluble solid content over time. The magnitude and rate of change were similar in dry matter and soluble solids. In contrast, although the pepper’s weight loss, dry matter, and TSS content all increased over time, the magnitude and rate of change were different in these variables. The daily weight loss had a more pronounced rate of change initially, which subsequently decreased from day 14 onwards. Conversely, the DMC_i_ and TSS content exhibited a more constant increase over the storage period.

**Figure 2:**
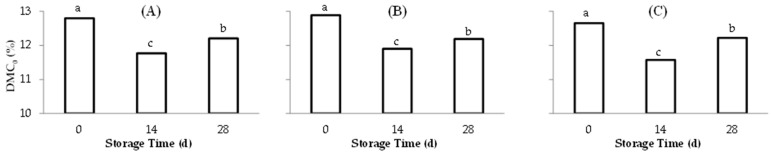
Dry-matter fruit content (DMC_0_, %), calculated as the percentage of dry matter that the fruit would have at harvest time and considering the hypothesis that any weight loss during storage is exclusively due to water loss. The results correspond to the average of: (**A**) all the conical and mini-conical cultivars; (**B**) exclusively for mini-conical cultivars; (**C**) and conical cultivars, according to the analysis of variance based on the model Y_ij_ = µ + α_i_ + ε_ij_. Different letters denote statistical significance for *p* < 0.05 according to the Bonferroni test.

The dry matter content (DMC_0_, %) of fruit stored for 0, 14, or 28 d at harvest was calculated to determine if the fruit underwent a loss in dry matter during the storage process compared to the content it would have had when harvested. The analysis was performed on all conical and mini-conical cultivars (Figure 2A), mini-conical cultivars (Figure 2B), and conical cultivars (Figure 2C). This calculation assumed that any weight loss during 14 or 28 storage days was solely due to water loss. If the hypothesis that all weight loss is due to water loss alone was correct, then the DMC_0_ values at 0, 14, and 28 storage days would be equal. However, the DMC_0_ values at days 14 and 28 were considerably lower than the DMC_0_ values at collection time. This indicated that dry matter loss accounted at least partly for the weight loss observed during storage. This effect was evident in all comparisons between cultivars, including when compared together (Figure 2A), when only the mini-conical cultivars were compared (Figure 2B), when only the conical cultivars were compared (Figure 2C), and even when each cultivar was individually compared for each month or jointly over the period studied (data not presented to simplify the results).

Conversely, assuming that the decrease in the dry matter of the fruit was constant and uniform throughout the storage period, then the DMC_0_ results on days 14 and 28 would exhibit a linear decrease as the storage time increased. However, in all the analyses, the DMC_0_ values at 14 d were consistently lower than the DMC_0_ values at 28 d. This behavioral pattern indicated that the greatest dry matter loss occurred during the first 14 days of storage. Additionally, considering that the DMC_0_ at 28 d represents the decrease in solid matter over the entire 0–28-day period (Figure 2) and that the weight loss approximately doubled from day 14 to day 28 of storage (Table 1), the decrease in weight between days 14 and 28 (attributable to the fruit’s dry matter) is negligible compared to that recorded during the first 14 storage days.

### 3.2. Relationships between Storage Time (ST), FWL, DMCi, and TSS

To explore the relationships between ST, FWL, DMC_i_, and TSS content in conical and mini-conical seedless pepper fruit, a correlation analysis was performed. This analysis used the Pearson’s correlation coefficient (r), which provides a quantitative measure of the associations present in the data. The correlation coefficients were calculated using all the data collected throughout the ST from cultivars ‘SL001’, ‘SL002’, and ‘SL003’ in the case of mini-conical-type peppers, and from cultivars ‘109’ and ‘563’ for the conical-type peppers.

The correlation matrix shown in Table 2 revealed a marked positive trend among all the variables studied for both pepper varieties (conical and mini-conical). Although all the correlations were statistically significant, the Pearson correlation coefficient values ranged from 0.45, which reflects the relationship between the ST and the soluble solids, to considerably higher values, such as 0.95 and 0.97, which indicate the close connection between the dry matter and soluble solid contents in the conical and mini-conical peppers, respectively. Notably, high correlation coefficients between fruit weight loss (0.90) and ST (0.92) in both pepper types were observed. These correlations underscore the influence of time on fruit weight loss, regardless of the cultivar.

**Figure 3:**
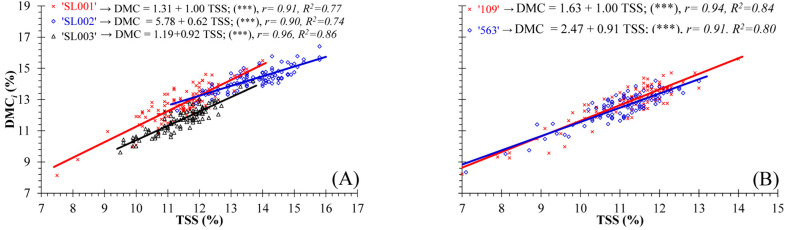
Models representing the relationships between the fruit’s dry matter content (DMC_i_, %) and total soluble solids content (TSS, %) at harvest time for fruit from: (**A**) cultivars ‘SL001’, ‘SL002’, and ‘SL003’; (**B**) cultivars ‘109’ and ‘563’. R^2^, is the coefficient of determination; r, the correlation coefficient; significant for *** *p* ≤ 0.001.

As shown in Table 2, the most prominent correlation was observed between the TSS and DMCi—two highly relevant variables in this study. The interdependence between the DMCi (%) and TSS content (%) at fruit harvest time is shown in Figure 3. This graphical representation indicates the independent models of fruit belonging to the mini-conical cultivars ‘SL001’, ‘SL002’, and ‘SL003’ (Figure 3A) as well as the models corresponding to the conical cultivars ‘109’ and ‘563’ (Figure 3B). The models developed using the data obtained for each cultivar at harvest exhibited statistically significant linear relationships. Furthermore, the Pearson correlation coefficients (r) and coefficients of determination (R^2^) associated with these models were positive and close to one (r ≥ 0.90 and R^2^ ≥ 0.77, respectively). The close relation between DMCi and TSS suggests that the phenomena and behaviors associated with post-harvest in fruit with high DMCi can be applied to fruit with high soluble solid values, and vice versa.

### 3.3. Relationship between the Fruit’s Commercial Life and Weight Loss during Storage

Table 1 shows the increase in the weight loss of the peppers over their storage period, indicating an average daily increment of 1.1%. Notably, during the first seven days, when the fruits were stored under cold conditions, the weight loss rate was considerably lower at 0.7% per day.

**Figure 4:**
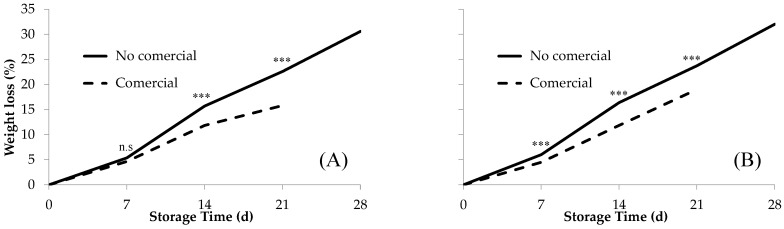
Evolution of fruit weight loss (%) for the commercially and non-commercially viable fruit as a function of storage time. The results correspond to the average for the (**A**) mini-conical cultivars; (**B**) conical cultivars, based on the analysis of variance, according to the model Y_ij_ = µ + α_i_ + ε_ij_ performed at 7, 14, and 21 storage days. n.s., not significant; significant for *** *p* ≤ 0.001.

The evolution of fruit weight loss (%) was calculated for commercially and non-commercially viable fruit to determine the effect of fruit weight loss on the commercial life of fruit. The weight loss value during storage was calculated as the average of the mini-conical (Figure 4A) and conical (Figure 4B) cultivars. For the same time and storage conditions in both cultivar types, the fruit weight loss value was significantly greater in fruit that were not fit for the market than in those that were fit. Moreover, this difference progressively increased during the storage period. For example, in conical peppers (‘109’ and ‘563’), the average weight loss after seven storage days was 1.5% higher in the non-commercially viable fruit than in the viable fruit. After 14 days of storage, this difference increased to 4.5% and reached a maximum of 4.7% after 21 d. At 0 and 28 days of storage, this difference could not be estimated because at harvest time (0 d), all fruit were marketable, whereas at 28 storage days, none of the fruit were marketable. A similar behavior was observed in the mini-conical cultivars, which reached a maximum difference of 6.8% in weight loss after 21 days of storage.

### 3.4. Dry Matter Content in Peppers as an Indicator of Their Commercial Life

The marketability of seedless pepper cultivars was initially calculated based on ST (d) to determine whether the dry matter content of peppers at harvest time can be used as an indicator of their greater or lesser commercial life. Subsequently, the effect of dry matter content on the commercial life of peppers was studied by jointly analyzing all fruit of the cultivars. Finally, the relationship between the dry matter content of the peppers and weight loss during their commercial life was evaluated.

The loss of commercial quality was determined for each fruit individually, taking into account senescence (decay), cold damage, and the presence of rot caused by microorganisms. Pathogen damage was caused by *Alternaria* sp., *Botrytis cinerea*, and *Erwinia carotovora* spp. The incidence of these rots was very low (<1%), so the total pathogen damage was considered without differentiating the type of pathogen.

Multiple binary logistic regression analyses of the ST (d) and the conical- and mini-conical-type pepper cultivars were performed to study the commercial life of each pepper phenotype. The models’ α and β coefficients were estimated, along with their statistical significance (*p* < 0.05), and the odds ratios for each coefficient, which indicate the effect of the predictors (ST and cultivars) on the marketability probabilities (Table 3). The estimated models exhibited a good fit, as indicated by the performed goodness-of-fit tests (high values for the Wald statistic and omnibus test, with *p* < 0.05; Hosmer–Lemeshow tests with *p* > 0.05; R^2^ of Nagelkerke and R^2^ of Cox and Snell close to 1). The models’ α and β coefficients exhibited a significant fit for the ST and the cultivars (*p* < 0.05). This is evident as α ≠ 0, β ≠ 0, and Exp (β) ≠ 1 over the entire confidence interval for Exp (β). Additionally, 73.7% of the variability in the marketability probability was explained by the ST and 72.8% by cultivars for the mini-conical and 72 conical types, respectively. Therefore, it can be stated that ST and cultivar influence the probability of peppers being commercially viable and that the peppers’ marketability probability decreases as the ST increases (β < 0).

**Figure 5:**
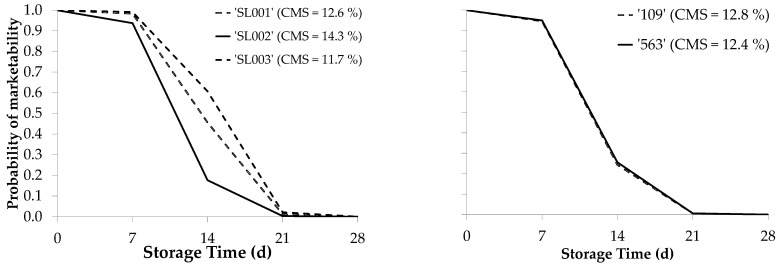
Average marketability probability of the seedless pepper cultivars used in the study.

Figure 5 shows the average marketability probability models for each pepper cultivar type shown in Table 3. Additionally, it indicates the average dry matter content associated with each cultivar at harvest time (day 0) used to determine its relationship with the shelf-life. In conical cultivars, the marketability probabilities and dry matter content were similar (12.8 and 12.4% for ‘109’ and ‘563’, respectively). In ‘mini-conical’ cultivars, ‘SL002’ had the lowest marketability probability and the highest dry matter content (14.3%), whereas ‘SL003’ had the highest marketability probability and the lowest dry matter content (11.7%), and the ‘SL001’ cultivar had an intermediate marketability probability and dry matter content (Table 3 and Figure 5).

**Figure 6:**
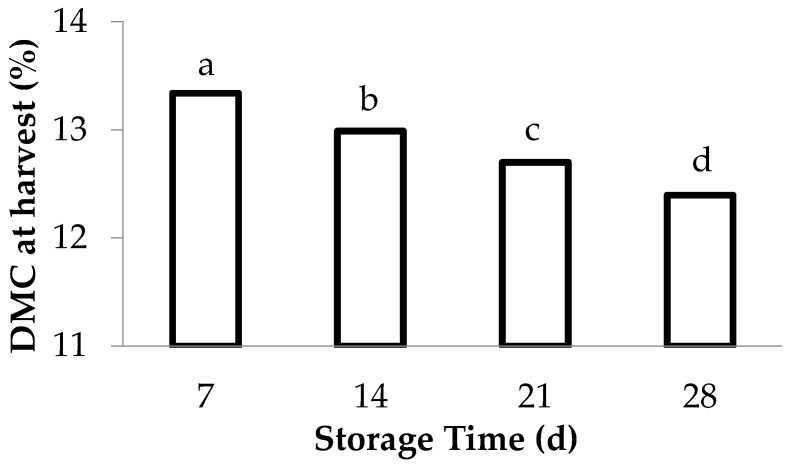
Dry matter content (DMC_i_, %) of peppers at harvest time depending on the days when the fruit are no longer marketable. The results correspond to the average of all the cultivars (conical and mini-conical) based on the analysis of variance, according to the model Y_ij_ = µ + α_i_ + ε_ij_. Different letters denote the statistical significance (*p* < 0.05) according to the Bonferroni test.

The results shown in Table 3 and Figure 5 suggest that cultivars with lower dry matter content have a higher marketability probability, and vice versa. Consequently, the effect of dry matter content on shelf-life was studied by jointly analyzing fruit from all cultivars (conical and mini-conical). Figure 6 indicates that peppers with a higher dry matter content at the time of harvest become unmarketable sooner than fruit with lower dry matter content. Specifically, fruit that were no longer marketable after 7 days of storage had an average dry matter content of 13.3% at the time of harvest. Fruits that were unmarketable after 14 days of storage had an average dry matter content of 13.0% at harvest, and those that ceased to be marketable after 21 days of storage had an average dry matter content of 12.7% at harvest. Finally, fruit that lost their commercial viability after 28 days of storage had an average dry matter content of 12.4% at harvest (Figure 6). Thus, a lower percentage of dry matter at harvest time may extend the marketability period of fruit during storage.

The dry matter values at harvest (categorized into discrete values of 11, 12, 13, 14, and 15%) were studied to determine the effect of evenly spaced increases in dry matter percentage at harvest time on the marketability probability. Multiple binary logistic regression analysis was performed considering the storage period (d) and dry matter content (%), which were considered as discrete variables. To obtain a holistic understanding of this behavior in seedless peppers, we included all conical and mini-conical cultivars in the statistical analysis.

**Figure 7:**
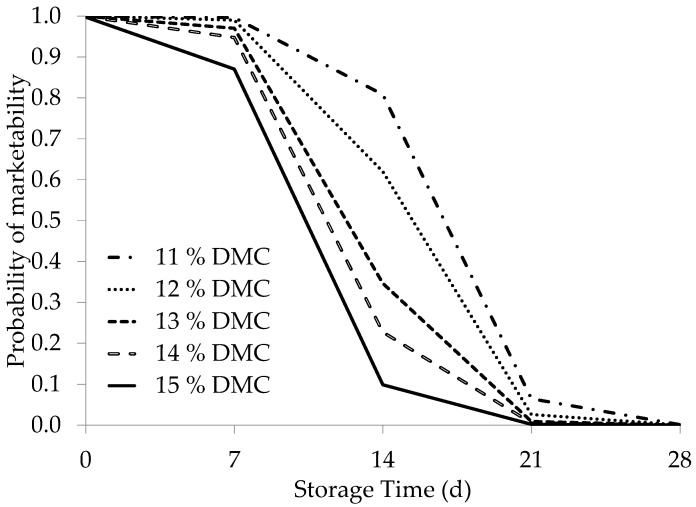
Marketability probability during storage based on the dry matter content (DMCi, %) of the fruit at harvest. The average results of all the cultivars (‘SL001’, ‘SL002’, ‘SL003’, ‘109’, and ‘563’) were included.

Figure 7 shows a graphical representation of the adjusted models. The logistic models had a consistent fit and the inherent α and β coefficients of the models showed a considerable fit with respect to the ST and dry matter content at fruit harvest time. These results show that as the dry matter content at the harvest time increased, the marketing capacity of the fruit decreased. A clear example of this phenomenon is shown when considering the 95% marketability probability in the curves shown in Figure 7. Here, the probability of 95% was reached after five storage days when the fruit had a dry matter content of approximately 15% at the harvest time. When the dry matter value at harvest was approximately 14%, the 95% marketability probability was achieved after seven storage days and after nine storage days when the average dry matter was 13%. Finally, when the dry matter content was 12 and 11% at the harvest time, this probability threshold was reached at 10 and 11 storage days, respectively.

**Figure 8:**
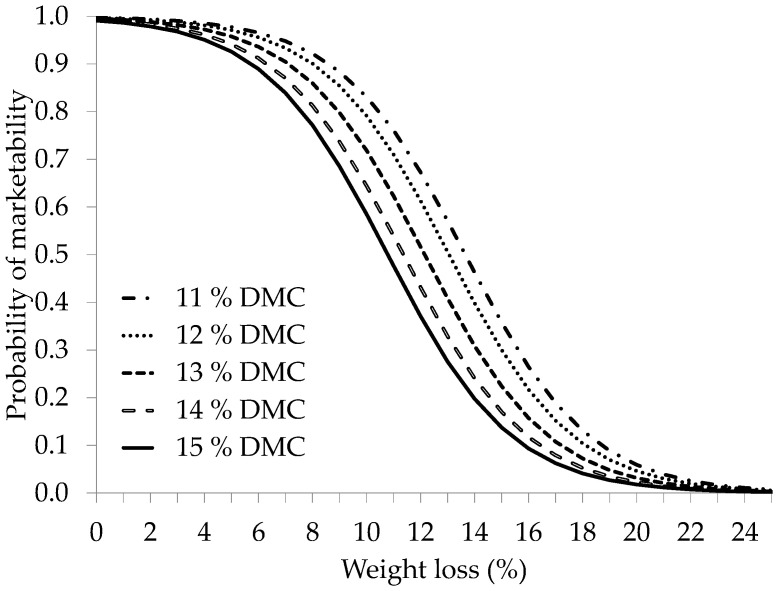
Marketing probability for the different dry matter percentages (DMC_i_, %) of the fruit at harvest, as a function of fruit weight loss. The average results of all the cultivars (‘SL001’, ‘SL002’, ‘SL003’, ‘109’, and ‘563’) are included.

After confirming that the fruit dry matter content at harvest affected its marketing capacity, the question arose as to whether this dry matter content influences fruit weight loss and vice versa. Thus, the marketability probability was studied by considering the dry matter content and fruit weight loss (Figure 8) as the influencing factors. The Hosmer and Lemeshow tests, omnibus analysis, and the R^2^ values of Cox and Snell and Nagelkerke were used to confirm the adequacy of the parameters’ fit quality in the obtained logistic regression models. The α and β coefficients assigned to the dry matter content and weight loss, respectively, were statistically significant (*p* < 0.000). These findings unequivocally indicate that marketability probability is influenced by the dry matter levels at harvest and weight loss during storage.

Figure 8 shows the influence of variations in weight on the probability of achieving successful fruit marketability, which, in turn, depends on the amount of dry matter present at harvest time. For example, a 90% probability can be achieved in two different scenarios: a 6% weight loss in fruit with 15% dry matter content at the time of harvest or a 9% weight loss in fruit with 11% dry matter content.

A different interpretation of these results is that the same decrease in weight affects the marketability probability differently, depending on the dry matter content of the fruit at harvest. For example, a 6% reduction in weight translates into a 97% marketability probability for fruit with 11% dry matter content, whereas for fruit with 15% dry matter content, the marketability probability is lower (89%). These results suggest that similar weight loss in the fruit affects the marketability likelihood more markedly when the dry matter content in harvest is high than when it is low.

Additionally, the results showed that fruit with a higher dry matter content at harvest were more susceptible to deterioration when they experienced weight loss during storage than fruit with lower dry matter content at harvest. According to the data presented in Figure 8, if some fruit with a dry matter content of 11% loses 10% of its weight during storage, its marketability probability decreases by 83%. Contrastingly, if the initial dry matter content is 15%, the marketability probability decreases further, dropping to 59%.

## 4. Discussion

This study investigated the post-harvest dynamics and the implications of certain parameters on the commercial shelf-life and quality of seedless conical and mini-conical pepper cultivars. Our findings suggested that the dry matter and soluble solid content of peppers at the harvest time and their evolution post-harvest may vary considerably between different cultivars belonging to the same type of parthenocarpic peppers. These results are consistent with those of other studies where similar patterns in terms of variations in dry matter and soluble solid contents were reported for various seeded pepper cultivars [6,29,30]. Díaz-Pérez et al. [31] reported that post-harvest weight and water loss of pepper fruit may differ between species and cultivars, which is in agreement with our results, which showed that fruit weight loss may vary among seedless pepper cultivars. The fruit weight loss caused water loss, which eventually resulted in a reduction in skin shininess. Wrinkling and fruit size reduction were triggered in all varieties studied when this loss was prolonged, resulting in fruit with a high probability of being rejected by consumers, which is in accordance with the findings of Finger and Pereira [8].

Kissinger et al. [9] provided detailed information on the relationship between cultivars and the rate of pepper water loss during storage. They found that the rate of water loss can markedly vary by 2–3 times between pepper cultivars. Most water loss occurs through the pericarpic surface rather than through the cut in the calyx [12]. Pepper fruit lack stomata [32] and thus, the cuticle is the main barrier to water loss. However, a definite correlation between the rate of water loss and cuticle thickness, cuticle content, or cuticle wax has not been demonstrated [33,34]. Our results, together with those reported by all these authors, suggest that selection of cultivars with a slow water loss rate—and, therefore, less weight loss—could improve the quality and shelf-life of pepper during storage.

As ST increased, so did the FWL, DMC_i_, and TSS content, both in seedless conical and mini-conical peppers. This was confirmed by the analyses of correlations and orthogonal contrasts, which showed the presence of significant positive linear effects. Additionally, the absence of quadratic effects indicates that changes do not follow a particular nonlinear pattern but rather follow a more linear progression as a function of time. The linear effect of weight loss as a function of ST was also evident in the results presented in studies on seed peppers, for example, in ‘California Wonde’, in the works of Moline and Hruschka [35] and Barzegar et al. [36], in red pepper [37], in green pepper [38], in hot pepper [39], and green bell pepper [40]. However, the linear effects of DMC_i_ and TSS content as a function of ST in parthenocarpic fruit have not been previously described.

Post-harvest fresh weight loss exhibits different intensities, which are associated with the pericarp thickness, V/S ratio, and composition of the waxy epidermis. Fruits with thicker pericarps are less susceptible to shrinkage caused by intense water loss [8]. During storage, both pepper types demonstrated a consistent increase in weight loss, dry matter content (DMC_i_), and soluble solid content as ST increased. However, the magnitude and rate of change differed between weight loss and the other two variables (DMC_i_ and TSS). Weight loss initially had a more pronounced change rate, whereas DMC_i_ and TSS content presented a more consistent increase.

Furthermore, when calculating the dry matter content (DMC_0_) of the fruit at harvest time and when the fruit was stored for 0, 14, or 28 d, we discovered that part of the weight loss that occurred during storage was due to the loss of dry matter. Additionally, the greatest dry matter loss occurred during the first 14 storage days, which was greatly reduced in subsequent days. Thus, post-harvest losses of fresh fruit may result both from water loss and the consumption of dry matter due to respiratory activity, with water loss being the most relevant. Additionally, long storage periods lead to greater daily water and dry matter losses at the beginning of the storage period, and the daily loss rate reduces during 10 to 14 days of storage. These differences suggest that the processes affecting weight loss and solid accumulation may not be identical and could be influenced by diverse factors, such as fruit composition, storage conditions, and the interaction between their components, as proposed by Finger and Pereira [8].

The relationships between ST, FWL, DMC_i_, and TSS content in seedless peppers have not yet been studied. Our results demonstrate a considerable positive correlation between these variables. Furthermore, these relationships were highly significant in the case of dry matter and TSS contents. Additionally, this association was consistent throughout the post-harvest period when considering all fruit of the different pepper cultivars and when analyzing the cultivars individually. Therefore, when parthenocarpic peppers have low TSS levels, their DMC_i_ is reduced, and vice versa. Importantly, the significant relationship identified between DMC_i_ and TSS content suggests that the post-harvest phenomena and behaviors associated with fruit with high dry matter values are also manifested in fruit with high soluble solid levels, and vice versa. In agreement with our findings, Maalekuu et al. [41] and Lannes et al. [42] identified a considerable correlation between the dry matter content and total solid concentration in seeded peppers. However, contrary to our results, these authors failed to establish a correlation between weight loss and dry matter content or between weight loss and total solid content. Furthermore, Popovsky-Sarid et al. [25] found that the total soluble solids (TSS) content decreased in seeded peppers with lower weight loss after storage.

Once the fruit is harvested, transpiration and water loss have considerable effects on its quality and shelf-life [17]. Studies on seeded pepper cultivars have shown that fruit weight loss is an indicator of post-harvest water loss [12,25]. Moreover, the greater the water loss, the greater the weight loss in peppers during storage [43]. Respiration, transpiration, and evaporation can cause water loss, which can lead to a decrease in fruit quality [44]. Specifically, this can result in extremely soft fruit, increase the incidence of rot, decrease their overall visual quality, and affect their taste and nutritional quality [45]. Therefore, weight loss is an important factor affecting the visual and overall quality of pepper fruit during storage [46]. However, these claims have not been substantiated for parthenocarpic pepper cultivars.

Our study on conical and mini-conical seedless peppers showed that weight loss during storage steadily increases over time. During the first few days in cold conditions, the rate of weight loss per day was noticeably lower. However, this decrease in weight loss during the first few days was accelerated over the subsequent days at room temperature. Our results suggest that controlling weight loss during parthenocarpic pepper storage is crucial to prolong their shelf-life, which is in accordance with the results reported by Ilić et al. [44] for seeded peppers.

The weight loss during storage can affect the quality and appearance of peppers [45]. Our results showed that non-commercially viable fruit experienced greater weight loss than commercially viable fruit. This disparity was accentuated as the ST progressed, indicating that post-harvest weight loss can be used as an indicator of quality during storage. These results are consistent with those described by Popovsky-Sarid et al. [25], who demonstrated a correlation between weight loss and post-harvest water loss in pepper fruit and suggested that water loss is an important factor affecting their quality and shelf-life. Similarly, Maalekuu et al. [41] confirmed the high correlation between the overall appearance of peppers during storage and weight loss, which was accompanied by a considerably robust correlation with their deterioration. Our findings offer valuable information for quality management and decision-making regarding the marketability of peppers during storage. Furthermore, it provides insights on time factors and storage conditions which are valuable for planning and implementing commercial strategies related to parthenocarpic peppers.

Additionally, our results revealed a significant relationship between the dry matter content of peppers at harvest time and the subsequent fruit durability in terms of marketing probability. This factor is a crucial indicator of their greater or lesser conservation capacity. Additionally, the high correlation found between the TSS content and DMC_i_ suggests that the TSS content of the fruit at harvest time could be considered as an indicator of the commercial life of the fruit during storage. DMC_i_ and TSS content have been used as indicators by other authors studying seeded pepper after harvest. For example, Díaz-Pérez et al. [31] used dry weight and TSS content in seeded peppers as indicators of post-harvest fruit quality under different shade levels in greenhouses. Hanaei et al. [47] used TSS to study a coating incorporating salicylic acid and caraway oil to reduce post-harvest cold damage in sweet peppers. Popovsky-Sarid et al. [25] stated that TSS content is an indicator of pepper fruit quality, which allows the identification of molecular markers linked to quantitative trait loci (QTLs) that control water loss in seeded pepper fruit post-harvest.

Additionally, our results showed a relationship between the commercial life of different cultivars and their average dry matter content. Therefore, the ST and cultivar type are crucial factors that affect the probability of a pepper being marketable. Cultivars with the lowest marketability probability had a higher dry matter content at harvest time, whereas those with the highest probability had a lower dry matter content. Cultivars with an intermediate marketability probability exhibited intermediate dry matter content. Consequently, cultivars with fruits that have higher dry matter content would be more suitable for local consumption, storage for short durations, or in the dehydrated pepper industry because less water needs to be removed during processing, thus reducing dehydration costs. Conversely, peppers with a low dry matter content are more appropriate for fresh consumption after prolonged storage, owing to their greater resistance to deterioration caused by dehydration. Furthermore, they have a prolonged fresh appearance, which is consistent with the findings of Lannes et al. [42].

We jointly analyzed all fruit of the conical parthenocarpic cultivars to assess the effect of dry matter content on commercial life and found that the higher the dry matter content of the pepper at harvest time, the less marketable they were compared to those that had less dry matter. These results suggest that the lower the percentage of dry matter at harvest time, the longer the period during which peppers can retain their commercial potential during storage. The inverse relationship between marketing probability and dry matter content suggests that low dry matter content is a desirable characteristic for peppers that need to be stored over a longer period. Díaz-Pérez et al. [31] concluded that seeded pepper fruit grown under shaded conditions exhibited a decrease in their dry matter and soluble solid content as the crop shading degree increased. However, fruits that were exposed to higher shading levels, and therefore had a lower solid content, exhibited greater post-harvest durability than those that were exposed to lower shading levels. These findings were similar to our results, as we also identified that fruits with lower dry matter and soluble solids contents had a longer shelf-life.

Finally, our results suggest for the first time that high dry matter and soluble solid contents in peppers are associated with low weight loss during storage. We determined that fruit with a high dry matter concentration at harvest time, which translates into a greater amount of TSS, are more likely to suffer visible deterioration during storage when they experience weight loss. In contrast, fruit with a higher water proportion than dry matter at harvest can tolerate greater water loss during storage without any visible spoilage signs, which compromises their commercial viability.

## 5. Conclusions

The dry matter and soluble solid content of peppers at harvest time can vary among different cultivars within the same type of parthenocarpic pepper. These parameters, together with fruit weight loss, can evolve differently among different cultivars during the post-harvest period.

Variations in FWL, DMC_i_, and TSS in seedless conical and mini-conical peppers increased with ST, indicating diversity in conservation responses between different cultivars. Additionally, weight loss was positively correlated with non-commercial viability of fruit, a phenomenon that increased with ST.

The daily weight loss, dry matter content, and soluble solid content in conical and mini-conical peppers increased over the storage period, following a similar pattern. The rate of change between dry matter and soluble solid content was consistent throughout the storage period. However, the initial rate of daily weight loss was more pronounced, which decreased after several storage days. Post-harvest losses of fresh seedless peppers resulted both from water loss and dry matter consumption due to respiratory activity, with water loss being the most relevant. Dry matter consumption in the stored fruit was greater during the first few storage days and subsequently decreased after several days.

A positive relationship was observed between ST, FWL, DMC_i_, and TSS in seedless peppers, which was more profound in the case of DMC_i_ and TSS content, suggesting that higher dry matter and soluble solid contents in seedless peppers at harvest time may be associated with less weight loss during storage. Additionally, fruits with lower dry matter percentages at harvest time maintained their commercial quality over a longer storage period. Therefore, a lower dry matter content at harvest may be a desirable characteristic of peppers intended for prolonged storage.

The dry matter and soluble solid content at harvest time, together with weight loss during storage, could be used as indicators to identify parthenocarpic peppers with longer or shorter shelf lives. To reduce waste produced from harvest to consumption, these indicators can be used to identify cultivars in breeding programs that have greater potential for commercial use, optimize the supervision of fruit batches destined for export, and provide a basis for developing quality control systems and commercial durability through non-invasive measurement methods.

Our study’s primary innovation lies in analyzing weight loss and dry matter changes during the storage of seedless peppers, an aspect previously unexplored. However, other important freshness parameters, such as changes in texture, sensory, and chemical parameters, are not included. It is essential to conduct further research on seedless peppers that addresses these additional parameters.

## Figures and Tables

**Table 1:** Fruit weight loss (FWL), dry matter content (DMC_i_), and total soluble solids content (TSS) during the storage of conical and mini-conical seedless peppers.

	“Mini-Conical” Type				“Conical” Type		
	FWL (%)	DMC_i_ (%)	TSS (%)		FWL (%)	DMC_i_ (%)	TSS (%)
A: Cultivar ^(&)^				A: Cultivar			
‘SL001’	16.0 b	13.6 b	12.2 b	‘109’	17.8 a	14.6 a	12.3 a
‘SL002’	14.9 c	17.4 a	16.5 a	‘563’	20.0 a	14.0 a	12.3 a
‘SL003’	23.5 a	12.7 c	11.9 c				
Significance	***	***	***	Significance	n.s.	n.s.	n.s.
B: Storage time (days)				B: Storage time (days)			
0		12.9 c	12.2 c	0		12.7 c	11.1 c
7	4.6 d			7	4.4 d		
14	14.3 c	14.1 b	12.9 b	14	15.2 c	13.6 b	11.7 b
21	22.3 b			21	23.8 b		
28	30.6 a	16.8 a	15.6 c	28	32.3 a	16.8 a	14.3 c
Significance	***	**	**	Significance	***	*	*
Orthogonal contrasts				Orthogonal contrasts			
Linear effect	**	*	*	Linear effect	**	*	*
Quadratic effect	n.s.	n.s.	n.s.	Quadratic effect	n.s.	n.s.	n.s.
A × B	***	***	***	A × B	***	n.s.	n.s.

The model for the analysis of variance was *Y_ijks_* = *μ* + *α_i_* + *β_j_* + (*αβ*)*_ij_* + γ*_k_* + (*α*γ)*_ik_* + (*β*γ)*_jk_* + (*αβ*γ)*_ijk_* + ε*_ijks_*. n.s. denotes no significance, while *, **, and *** significance at a *p* ≤ 0.05, 0.01, and 0.001 level, respectively. Numerical values followed by different letters denote statistical significance for *p* < 0.05 according to the Bonferroni test. ^(&)^ ‘SL001’: red fruit cultivar, ‘SL002’: orange fruit cultivar, and ‘SL003’: yellow fruit cultivar. The linear and quadratic effects for the storage time were calculated using orthogonal contrasts.

**Table 2:** Correlations between the storage time (ST), fruit weight loss (FWL), dry matter content (DMC_i_), and total soluble solids content (TSS) in conical and mini-conical-type peppers.

	“Mini-Conical” Type				“Conical” Type			
Variable	ST (d)	FWL (%)	TSS (%)	DMC_i_ (%)	ST (d)	FWL (%)	TSS (%)	DMC_i_ (%)
ST (d)		0.90 ***	0.45 ***	0.49 ***		0.92 ***	0.59 ***	0.63 ***
FWL (%)			0.70 ***	0.73 ***			0.72 ***	0.77 ***
TSS (%)				0.97 ***				0.95 ***
DMC_i_ (%)								

Pearson’s correlation coefficient (r) significant for *** *p* ≤ 0.001.

**Table 3:** Multiple logistic regression parameters used to calculate the marketability probability of seedless peppers as a function of the ST (d) and the cultivars.

	Coefficients			Odds Ratio	95% CI for [Exp(β)]	
Variables	(α, β)	Wald χ^2^	*p*	(Exp (β))	Inferior	Superior
Constant	8.901	501.55	<0.000			
Storage time (d)	−0.605	527.46	<0.000	0.546	0.519	0.576
Cultivar ^(i)^						
‘SL001’	−0.311	3.92	0.0474	0.733	1.023	1.900
‘SL002’	−1.673	89.83	<0.000	0.188	3.826	7.666
‘SL003’		Reference				
Constant	6.904	342.262	<0.000			
ST (d)	−0.570	420.036	<0.000	0.566	0.536	0.598
Cultivar ^(ii)^						
‘109’	−0.067	0.148	0.7009	0.935	0.664	1.317
‘563’		Reference				

CI: Confidence interval. (i) Deviation explained by the model = 73.7%. Likelihood-ratio test (omnibus; *p* < 0.000). Hosmer and Lemeshow test (*p* = 0.071), R^2^ of Cox and Snell: 0.636; R^2^ of Nagelkerke: 0.851. (ii) Deviation explained by the model = 72.8%. Likelihood-ratio test (omnibus; *p* < 0.000). Hosmer and Lemeshow test (*p* = 0.196). R^2^ of Cox and Snell: 0.635; R^2^ of Nagelkerke: 0.847.

## Data Availability

The original contributions presented in the study are included in the article, further inquiries can be directed to the corresponding author.
